# Simultaneous activation of border-associated immune cells and glial cells at the CNS-meningeal interface after subarachnoid haemorrhage in rats

**DOI:** 10.1007/s00429-026-03156-y

**Published:** 2026-07-22

**Authors:** Thannoon Masood, Szandra Lakatos, Melissza Ignácz, Judit Rosta

**Affiliations:** 1 Department of Neurosurgery, Albert Szent-Györgyi Medical School, University of Szeged, Semmelweis utca 6, Szeged, 6725 Hungary; 2 Department of Physiology, Albert Szent-Györgyi Medical School, University of Szeged, Dóm tér 10, Szeged, 6720 Hungary

**Keywords:** Meningeal immunity, Neuroinflammation, Border-associated macrophage, Subarachnoid haemorrhage, Meninges, Mast cell, Neuroinflammation

## Abstract

Border-associated macrophages (BAM) and mast cells are resident immune cells at the peripheral CNS borders, strategically located close to the brain surface, potentially influencing the homeostasis of the underlying parenchyma. Subarachnoid haemorrhage (SAH), when blood enters between the meningeal layers that cover the brain, is associated with neuroinflammation, which has been shown to play a critical role in subsequent brain damage; however, the impact of the activation of border-associated immune cells on the pathomechanism of the disease has not been investigated. Our aim was to examine inflammatory reactions that occur simultaneously at the cellular level in various compartments of the CNS: meningeal, subdural space, and parenchyma after experimental SAH in rats. Using immunohistochemistry, we performed the morphological characterisation of the BAM subpopulations in meningeal preparations. Additionally, confocal microscopy and image analysis were used to evaluate the reactive state of microglia cells and the integrity of the glial boundary in the upper fronto-parietal cortex of the rat 72 h after SAH. We demonstrated morphological alterations of BAM populations in the meninges and parallel disintegration of the astrocyte barrier due to haemorrhage. Furthermore, we confirmed the crucial role of mast cells in subsequent glial reactions. Our results suggest that activation of border-associated immune cells, contemporaneously with the early neuroinflammatory reactions that take place in the brain parenchyma, proposes a feasible signalling between these compartments following haemorrhage. Further studies are to be performed to reveal the importance of CNS meningeal border as a communication interface in the pathomechanism of SAH.

**Supplementary Information:**

The online version contains supplementary material available at https://doi.org/10.1007/s00429-026-03156-y.

## Introduction

Neuroinflammation is defined as the inflammatory response of the central nervous system (CNS) to altered homeostasis. In recent years, neuroinflammation has been identified to play a critical role in several diseases, such as stroke, Alzheimer’s disease, and epilepsy, for which diseases inflammation otherwise is not the primary aetiology (Arvin et al. 1996; Yokota et al. 1995; McGeer and McGeer 2002). Astrocytes and microglia, the most prominent glial cells in the CNS, are key components of inflammatory reactions as they react to insults affecting the CNS by modulating their morphological and functional characteristics through a process called ‘reactive gliosis’. Microglia, as resident immune cells, uninterruptedly monitor the microenvironment through their ramified processes and exert a protective function by phagocytosing pathogens. Microgliosis refers to reactive proliferation, migration, and phenotypic switch of microglia in response to pathological signals (Herber et al. 2006). Astrocytes, the most abundant type of glial cells, possess a wide range of homeostatic functions, including buffering excess neurotransmitters or regulating synaptic and blood-brain barrier (BBB) functions. Their end feet are in contact with the vasculature (Hösli et al. 2022), therefore astrocytes are promoted to sense the brain microenvironment and also peripheral signals in the blood. At the parenchymal surface, astrocyte foot processes are associated with the parenchymal basal lamina forming the glia limitans. However, the glial membrane is not an absolute barrier, but can adequately limit the diffusion of substances into the brain tissue (Nau et al. 2010). Consequently, changes in astrocyte function or morphology occurring in astrogliosis impact the integrity of the barrier.

In fact, the vast majority of CNS disorders are associated with the disruption of the BBB. Fragmentation of these barriers together with the expression of adhesion molecules in endothelial cells has been shown to facilitate the infiltration of circulating immune cells into the brain parenchyma after ischemic stroke (Brait et al. 2012; Chu et al. 2014; Marchetti and Engelhardt 2020). In the last decade, it has been accepted that the brain is not completely immune-privileged and that elements of the CNS are sensitive to inflammatory events that occur in the periphery (Engelhardt et al. 2017). Neuroinflammation can involve reactive components of the CNS and also elements recruited from the periphery (Berriat et al. 2023). It has been suggested that the distinct arrays of immune cells confined to the border region of the CNS mediate the diversity of immune responses in the brain (Merlini et al. 2022; Smyth and Kipnis 2025). Border-associated macrophages (BAM) are resident immune cells at the peripheral interface of the CNS, located strategically to be close to the parenchymal surface, therefore BAMs can potentially influence the homeostasis of the underlying parenchyma (Gerganova et al. 2022). Meningeal BAMs (mBAMs) are located in the pia and dura mater of the meninges, where they survey the surrounding tissue and display morphological plasticity, and can be induced to adopt different migrational states. In the pia mater, BAMs display simple morphology and higher motility compared to microglia, while dural mBAMs are elongated and exhibit a lower motility rate under resting conditions (Kierdorf et al. 2019). Studies have shown migration and accumulation of BAMs in ischemic tissues (Pedragosa et al. 2018; Holfelder et al. 2011; Rajan et al. 2020a), as well as a shifted distribution and altered phenotype of mBAM subsets after stroke (Rajan et al. 2020b). In addition to BAMs, mast cells (MC) residing in large number in the dura mater can respond to a wide variety of stimuli and by releasing inflammatory mediators play a pivotal role in the pathogenesis of neuroinflammation (Hendriksen et al. 2017; Strbian et al. 2006; Rustenhoven et al. 2021). The role of MCs in BBB impairment has not been fully elucidated, but deficiency or inhibition of MCs resulted in reduced BBB permeability, proposing the impact of MCs on the disruption of the BBB (Esposito et al. 2001; Yue et al. 2023; Ribatti 2015).

Neuroinflammation has been recognized as critically involved in both early and delayed brain damage after subarachnoid haemorrhage (SAH) (Schneider et al. 2018; Mathiesen et al. 1993; Heinz et al. 2021; Luo 2023) when blood enters the subarachnoid space between the arachnoid and pia mater. Severe neurological complications with recovery of varying degrees are associated with SAH and the best treatment for these neurologic complications is yet to be clearly defined. The concept that the presence of blood between the meningeal layers proposes the activation of inflammatory cells has not been thoroughly researched. To date, research on SAH has focused on early brain injury and neuroinflammation after haemorrhage, but the approaches used within these studies have not provided information about the inflammation of the meninges.

In this study, we have examined the inflammatory changes that occur at the cellular level in various compartments of the CNS: meningeal layers and parenchyma and we assume signalling between these compartments. We have performed morphological characterisation of BAMs in leptomeninges and also dura mater encephali after SAH. We further provide morphological evidence for the disintegration of the astrocyte barrier after haemorrhage and have confirmed the crucial role of mast cells in subsequent glial reactions.

## Methods

### Surgical procedure and SAH induction

**Fig. 1:**
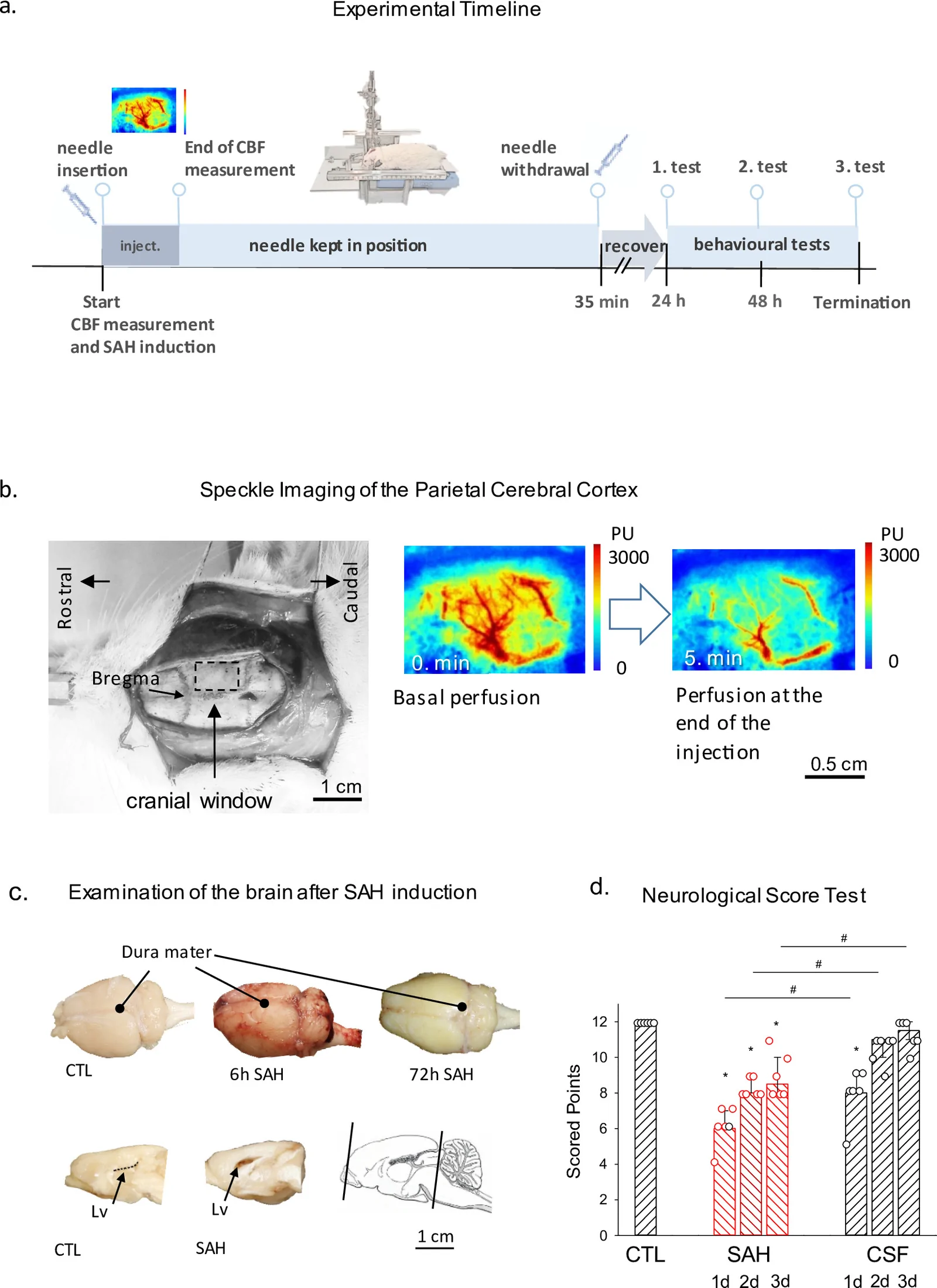
Basic characteristics of the SAH model. **a** The experimental timeline shows the insertion of a needle and the injection of blood into the intracisternal cavity to induce SAH, the parallel measurement of the cerebral perfusion (CBF), and the survival time. **b** Decreased cerebral perfusion as a result of intracisternal injection (inject.). On the left, a photograph indicates the location of the cranial window (dotted square) prepared for the measurements taken on the parietal skull of the rat. Scale bar: 1 cm Speckle images of the parietal cerebral cortex before (0 min) and at the end (5 min) of intracisternal injection of blood. Cerebral perfusion was monitored during the 5 min injection interval. The corresponding quantitative colour-coded perfusion maps with their respective colour scales (right column) show a decrease in the perfusion at the end of the injection (5 min) compared to the basal value (0 min) (right); scale bar: 0.5 cm. **c** Examination of the brain after SAH induction. Upper panel: The brains were dissected with the dura matrix kept intact after transcardial perfusion with saline in the control (CTL, left) and 6–72 h after SAH induction (6hSAH, 72hSAH, respectively). Marked blood deposition under the dura mater throughout the entire cerebral surface can be readily observed in SAH animals, whereas blood is absent in CTL. The route of the injected blood was verified by macroscopic examination of the rat brain. The presence of blood was demonstrated 6 h after SAH (6hSAH); however, there was no visible bleeding or residual blood in the subdural space in the parietal brain area 72 h after haemorrhage (72hSAH). Bottom panel: 72 h after SAH induction the lateral ventricle is enlarged as observed after transcardial perfusion with saline (right, medial view of the right hemisphere) unlike in CTL (left). Lv: Lateral ventricle. **d** Intracisternal injection of blood worsens the neurological outcome. The overall results of the four behavioural tests of the control animals (CTL) and cerebrospinal fluid injected (CSF) animals were significantly better than those of the haemorrhage group (SAH). All values are expressed as mean ± S.D. Data were analysed with ANOVA followed by the Fisher post hoc test. *n* = 6 in each group. A probability level of *p* < 0.05 was considered statistically significant. *: *p* < 0.001 versus the CTL group, #: *p* < 0.001 versus CSF group

An intracisternal single injection rat model was used to induce experimental SAH (Wajima et al. 2017; Masood et al. 2024). For surgical procedures general anaesthesia was induced with isoflurane (4%) (R546Pro, Gas Evacuation Apparatus, RWD Life Science, Shenzhen International Innovation Valley, Guangdong, China). Rats were placed in a stereotactic frame, the skin was incised in the cervical region, and the atlanto-occipital membrane was exposed after muscle dissection. During the operation, 200 µL of autologous blood (group SAH) was injected into the cisterna magna with a 26 gauge needle for a period of 5 min (Solomon et al. 1985) (Fig. 1a). After injection, the needle was held in place for another 30 min to prevent the leakage of cerebrospinal fluid (CSF). 30 min after injection, the skin was closed with sutures and the animals were allowed to recover. A group of animals was injected with the same volume of artificial cerebrospinal fluid (aCSF, composed of 119 mM NaCl, 26.2 mM NaHCO_3_, 2.5 mM KCl, 1 mM NaH_2_PO_4_, 1.3 mM MgCl2, 10 mM glucose) (CSF group). Untreated control naïve animals were used as controls(CTL group). Physiological parameters were recorded throughout the surgical procedure by measuring blood pressure (mean 105 ± 15 mm Hg; Coda M1 Monitor Noninvasive Blood Pressure System, Kent Scientific Corporation, Torrington, CT 06790, USA), blood oxygen saturation (pulse oximetry, SpO2 75–95%; Pulox PO-600VET, Novidion Gmbh 51149 Köln, Germany) and body temperature (37 ± 0,5 °C, Supertech TMP-5a, Pecs H-7624 Hungary). The rats were returned to their home cages after complete recovery from anaesthesia. To minimise the suffering of the animals, Buprenorphin (0.05 mg/kg) was administered subcutaneously before and after the surgical procedure. Seventy-two hours after the induction of SAH/injection of aCSF rats were transcardially perfused and their brain and dura mater were removed for histochemical analysis.

Relative cerebral blood flow (CBF) monitoring was performed with laser speckle imaging (RFLSI-ZW laser speckle contrast imaging system, RWD Life Science, Shenzhen International Innovation Valley, Guangdong, China) throughout the intracisternal injection to verify proper SAH induction, as indicated by a sharp reduction in CBF (Fig. 1c). For CBF measurements, the skull bone was exposed and the right parietal bone was thinned with a drill to improve the visibility of blood vessels for laser perfusion measurements. Taking into account the probable effect of skull thinning on glial cells, 4 animals (2–2 rats for adequate injection of blood and aCSF) were used for cerebral perfusion measurements but not for histological studies.

### Degranulation of mast cells

A subset of rats was injected with compound 48/80 (C48/80) to deplete mast cells (C48/80 group). Mast cell degranulation was performed by injecting a single dose of 0.75 mg/kg of C48/80 (Sigma-Aldrich, Merck KGaA, Darmstadt, Germany) intraperitoneally (at a concentration of 1 mg/ml). C48/80 was administered according to a previously published protocol (Kaida et al. 2010), with a slightly reduced dose to minimize systemic inflammatory effects. To further limit systemic reactions, animals received antihistamine (cetirizine, 900 µg/kg as intraperitoneal injection) treatment. Mast cell degranulation was confirmed histologically, and a marked reduction in mast cell number was observed 4 days after treatment; therefore, a 4-day post-treatment survival time was used for subsequent analyses (C48/80 group). In a group of animals, four days after injection of C48/80, SAH was induced (C48/80-SAH group).

### Experimental animals

Thirty-four (35) adult male Wistar rats weighing 350 to 400 g were used. Thirty (30) rats were randomly assigned to one of the experimental groups: (1) untreated control animals (CTL, *n* = 6), (2) intracisternal injection of autologous blood (SAH, *n* = 6), (3) intracisternal injection of aCSF (CSF, *n* = 6), and (4) C48/80-pretreated animals (C48/80, *n* = 6). (5) In a subset of animals, C48/80-induced degranulation of mast cells was performed four days before SAH induction (C48/80 + SAH, *n* = 6). Furthermore, four (4) animals were used to verify the intracisternal injection method in the induction of SAH by monitoring cerebral perfusion during injection of blood or aCSF. Additionally, one (1) animal was used to check the presence of blood on the surface of the brain 6 h after induction of SAH (Fig. 1c). The size of the experimental groups was determined based on the calculation of the sample size using Power and Sample Size 3.043 software (power 80%, α 0.05). The experiments were approved by the Ethics Committee for Animal Care of the University of Szeged (approval ID: XIV./2973/2016) and carried out in accordance with Directive 2010/63/EU of the European Parliament. All efforts were made to minimise the number of animals used.

### Neurobehavioural assessment

We performed neurobehavioural tests to assess neurological functions 1, 2, and 3 days after surgery in the CTL, SAH, CSF, and C48/80 + SAH groups. Four functional behavioural tests were applied to evaluate limb symmetry, climbing, auditory (startle) reflex, and spontaneous activity, and the results of each test were graded from 0 to 3. Neurological condition was scored as follows: 3 – no deficit, 2 – slightly affected, 1 – severely affected, 0 – no function compared to control. The overall behavioural test score was calculated as the sum of the subscores (0–12).

### Tissue preparation and immunohistochemistry

For immunohistochemistry, 72 h after surgery, rats were anaesthetised with isoflurane (4%) and perfused transcardially with 4% paraformaldehyde in phosphate buffer (PB, pH 7.4), then the brain was carefully removed. The isolated brain was post-fixed for 2 h in paraformaldehyde (4%), then immersed in 0.1 M PB containing 25% sucrose at 4 °C for 20 h, and cut into 25 μm thick coronal sections using a cryostat (Leica CM1950-Kryostat, Leica Biosystems Nussloch GmbH, Nussloch, Germany). The inner meningeal layers (leptomeninges), the arachnoid membrane, and the pia mater adhere to the surface of the brain. To prepare the leptomeningeal preparation, a parietal part of the leptomeninges around the caudal rhinal vein was carefully removed from the surface of the brain by forceps and used as whole-mount samples. Subsequently, the parietal dura mater encephali was excised from the skull and used as a whole-mount sample.

The macrophage marker ionised calcium binding adaptor-1 molecule (Iba1), the CD11b (clone OX42, also known as integrin alpha-M) microglia marker (OX42), and the astrocyte marker glial fibrillary acid protein (GFAP) were used to visualise BAMs and parenchymal glial cells for morphological analysis. OX42 (CD11b) was used as a well-established marker for microglia, as it recognizes complement receptor 3 (CR3) expressed on resident CNS myeloid cells and has been widely applied for microglial visualization in rodent brain tissue. Although Iba1 also labels microglia, OX42 was selected due to its strong membrane-associated staining, which allows clear visualization of microglial morphology.

The coronal brain sections and meningeal preparations were then processed for indirect immunofluorescence staining using rabbit anti-Iba1 antibody (1:750, Abcam, Cambridge CB2 0AX, UK), mouse anti CD11b/c Monoclonal Antibody (OX-42) antibody (1:750, Thermo Fisher Scientific, Waltham, Massachusetts, USA) and rabbit anti-GFAP antibody (1:750, Sigma-Aldrich, Merck KGaA, Darmstadt, Germany) as primary antibodies. Secondary antibodies were immunoglobulins labelled with Cy3 and DayLight488 (1:500, Jackson Immunoresearch Laboratories, Westgrove, PA, USA).

### Image acquisition and immunohistochemical analysis

The imaging and analysis of the sections was performed with a ZEISS LSM 700 laser scanning microscope with Zen 2010 software (ZEISS, Oberkochen, Germany). Photos were captured with 20x magnification under the same exposure time and camera gain, with applying the same optical section step (1.2 μm) for all sections. For our data analysis, Z-stack images were processed to generate maximum projection images. The captured images were saved in 8-bit greyscale TIF format. For image analysis, ImageJ 1.54c27 software was used (Schneider et al. 2012). The ‘IsoData’ threshold setting was applied.

#### Immunohistochemical analysis of leptomeningeal and dura mater preparation

Dura mater preparates obtained from the cranial vault of each animal were used for immunohistochemical staining and analysis. A 1 mm^2^ area around the branches of the middle meningeal artery (we defined an area at a maximum distance of 100 μm from the visible arterial branches) was selected for analysis. Iba1, a widely used macrophage marker, was used to determine the total macrophage population. The quantification of Iba1-immunoreactive (Iba1-IR) cells was carried out using the ImageJ 1.54c27 software ‘Analyze Particles’ feature and their distribution (number of cells/200 × 200 µm area) was calculated in each section. For morphological analysis of Iba1-IR cells, three shape descriptors were determined: circularity, roundness, and elongation (the ratio of the major to the minor axis, AR). Circularity was determined using the formula: $\:Circularity=4\pi\:\left(\frac{Area}{{Perimeter}^{2}}\right).$ Circularity value of 1.0 indicates a perfect circle. Roundness (Round) values range from 0 to 1 and was calculated using the formula: $\:Roundness=\frac{4\:Area}{\pi\:{\left(Major\:Axis\:Length\right)}^{2}}$. According to the calculated shape descriptors, four shape categories were identified: (1) circular with high circularity, (2) elongated with low circularity but high AR value, and two intermediate stages, (3) the amoeboid with higher roundness, and (4) intermediate, similar to Travnickova et al. (2021). Circularity was used to distinguish round objects (circularity > 0.2). Then roundness and elongation factor were used to set apart non-round subjects into 3 subgroups: elongated, amoeboid and intermediate.

#### Immunohistochemical analysis in coronal brain sections

A cryostat section containing the frontoparietal motor cortex (Bregma-2.4–2.8 mm) randomly selected from each animal was analysed using ImageJ.

For morphometric analysis of the end feet, an intensively labelled region on the surface of the brain parenchyma was demarcated, and the intensity of the GFAP-labelling was determined as the optical density. Furthermore, the width of the GFAP-immunopositive layer (in µm) was determined in each animal. Ten lines 20 μm apart were drawn perpendicular to the stream of labelling along the entire length of the region of interest (1.8–2 mm laterally from the longitudinal fissure). The length of the ten lines was measured and their average was used as the reference value for each animal.

Activation of microglial cells was evaluated using the OX42 antibody as a specific microglial marker. A 200 μm x 200 μm square (1.8–2 mm laterally from longitudinal fissure and 200 μm depth in the brain parenchyma) as the region of interest was demarcated, and the intensity of the OX42-labelling was determined as optical density. Furthermore, for morpholometric analysis, twenty microglial cells per animal were traced using Sholl analysis and ramification as indicated by branching index (BI) was determined and quantified with ImageJ Fiji software. BI defines the number of intersections made of concentric circles relative to the distance from the soma. To evaluate branching tendency of individual microglial cells, BI was calculated using radius step size 2 μm.

### Histology, the quantification of mast cells

Quantification of meningeal mast cells was performed in five animals each from the CTL, SAH, CSF, and C48/80 groups. Dura mater whole-mount preparations were immersed in 0,5% Toluidine Blue solution (Sigma-Aldrich, Merck KGaA, Darmstadt, Germany) in acetic acid for 20 min, then washed and placed on slides. Tile scan images captured with 20x magnification with a ZEISS LSM 700 laser scanning microscope were used for our data analysis. An area of 500 × 500 μm was selected around the branches of the middle meningeal artery for analysis. The number of mast cells present in the selected region (number of cells/0.25 mm^2^) was determined three days after SAH or four days after intraperitoneal injection of C48/80.

### Statistics

All representative graphs were analysed and created in Statistica 13 (StatSoft, USA) or Sigmaplot 12.0 (SyStat software Inc., Grafiti, USA) software. After confirming normality and homogeneity of the variances, the data were analysed by the analysis of variance (one-way ANOVA) or ANOVA on ranks (Kruskal-Wallis). Values are expressed as median or mean ± standard deviation (SD). Statistical comparisons of the number of meningeal mast cells were performed between the CTL and C48/80 groups using Student’s t-test or the Mann-Whitney Runk sum test, where appropriate. In all tests, a probability level of *p* < 0.05 was considered statistically significant, the reported p values are two-sided.

## Results

### Baseline characteristics of the SAH model

First, to ensure SAH was induced properly, cerebral perfusion over the parietal cortex was monitored for 5 min of intracisternal injection of blood and aCSF in a subset of animals. A sharp reduction in cerebral blood flow (on average 18–20%) was measurable during the injection period in all animals tested, confirming the accuracy of the injection method. The measured physiological parameters suchs blood pressure (mean blood pressure 105 ± 15 mmHg) and blood oxygen saturation (SpO2 75–95%) remained within the normal range during the injection.

Twenty-four hours after induction of SAH, neurobehavioural tests were performed to evaluate the neurological outcome. The neurological scores showed significant SAH-induced neurological damage compared to the CTL group, with mean values of 6.5 ± 0.22, points vs. 12 points for the control. The observed neurological deficit did not recover in SAH animals during the next two days: with a total score of 8.5 ± 0.34, and 8.0 ± 0.36, on days 2 and 3 post-injection, respectively (Fig. 1d). A moderate reduction in neurological scores in the CSF group compared to the CTL group 24 h after the intracisternal injection of aCSF was observed. However, the animals were almost completely recovered in the following 72 h (Fig. 1d).

### SAH changes the morphological state of macrophages in the meningeal tissues

**Fig. 2:**
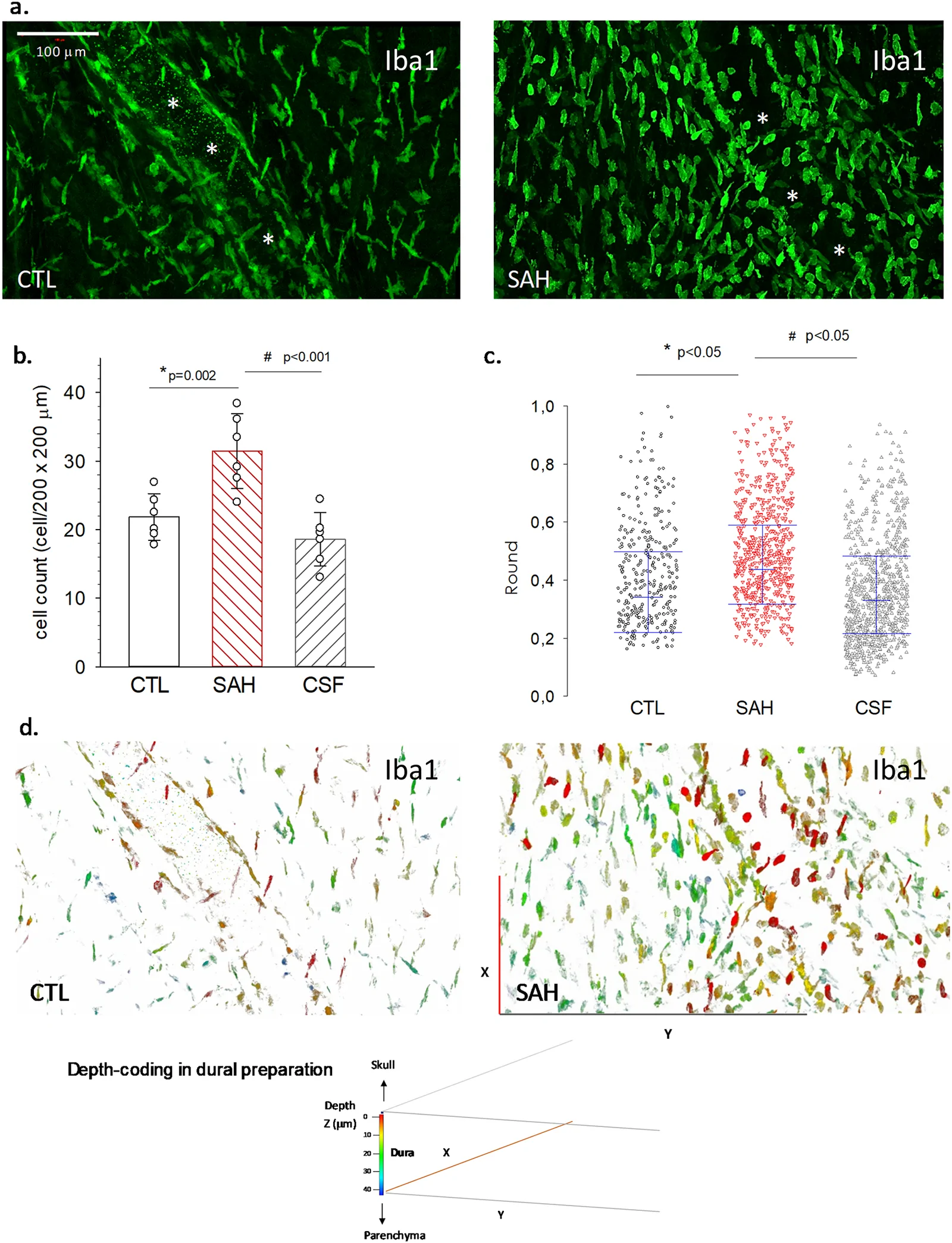
SAH impacts the macrophage population in the rat dura mater. **a** Representative images of meningeal macrophages stained with Iba-1 antibody - in control (CTL) and after SAH (SAH)- showing the presence of Iba-1-immunopositive cells around the middle meningeal artery. Asterisks (*) indicate the lumen of the blood vessel. Scale bar: 100 µm. **b** Cell counting data indicate an increase in the number of Iba1-immunopositive cells in the rat dura after SAH. Data are expressed as mean ± S.D. and were analysed by One Way ANOVA followed by Fisher’s post hoc test. *: significant vs. CTL; #: significant vs. CSF. n = 6 in each group **c** Morphometric analysis of meningeal macrophages indicates an increase in roundness of Iba1-immunopositive cells after SAH. (n = 311, 590 and 790, the number of cells that were analyzed in CTL, SAH and CSF group.). Data were tested by Kruskal-Wallis One Way ANOVA on Ranks and pairwise comparisons were performed with Dunn’s post hoc test. The graph shows the roundness (’Round’) of individual cells in the CTL (black circle), SAH (red triangle) and CSF (grey triangle) groups, and values are also expressed as median with range (IQR 25–75%) (blue lines) *: significant vs CTL; #: significant vs. CSF. **d** The depth coding of Iba1-immunopositive cells - in control (CTL) and after SAH (SAH)- indicates the localisation of macrophages at different depths of dural preparation by using a colour code. Z axis represents the cranial to ventral axis. of the dura mater. Images show an increased number of cells with circular morphology mostly in the upper tissue layer after SAH

Antibody against Iba-1, a macrophage marker was used to visualise mBAMs in dura mater whole-mount samples (Fig. 2a). The parietal dura mater surrounding the middle meningeal artery was selected due to the involvement of the perivascular dura in leukocyte recruitment and transmigration (Fig. 2B Supplementary). Immunohistochemical analysis showed increased number of Iba1-immunopositive cells in rat dura 3 days after SAH (22 ± 1.38 vs. 31 ± 5.48 cells/200 × 200 μm, CTL vs. SAH, *p* < 0.001) (Fig. 2b). As the morphology of macrophages particularly depends on the various activity states of the cells, we have evaluated the effect of bleeding on the morphology of mBAMs. Injection of CSF did not cause significant changes in the number or morphology of Iba1-immunopositive cells (CSF group compared to CTL animals: 19 ± 1.60 vs. 22 ± 1.38 cells/200 × 200 μm; round 0.33 vs. 0.34) (Fig. 2b, c). However, three days after SAH, a change in the shape distribution of mBAM was observed, characterized by a significant increase in circularity (0.25 vs. 0.27 CTL vs. SAH, *p* = 0.002) and roundness (0.34 vs. 0.44 CTL vs. SAH, *p* < 0.05, Fig. 2c) of cells positive for Iba1.

**Fig. 3:**
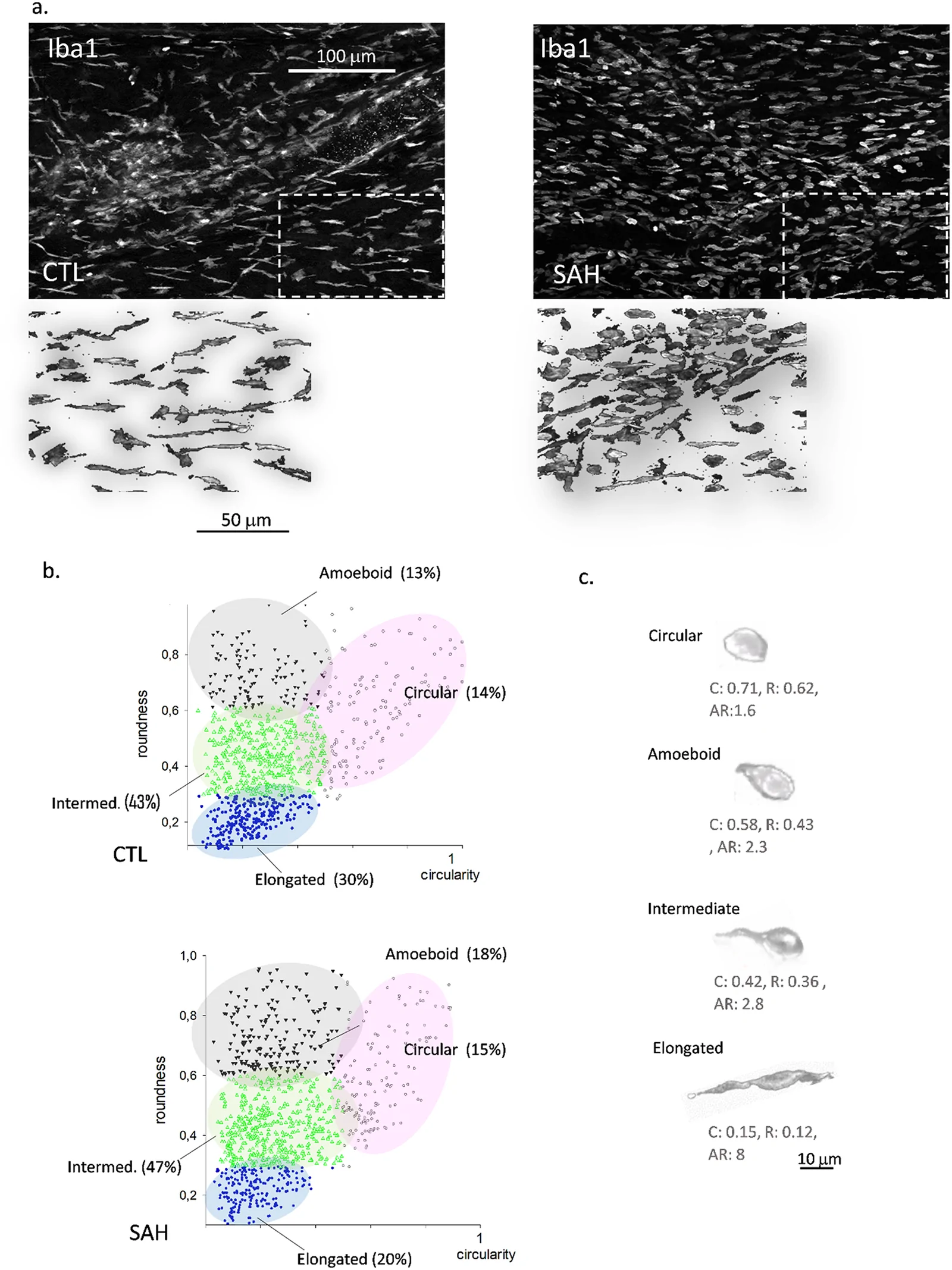
SAH induces a variation in the shape distribution of BAMs in the dura mater. **a** Representative photomicrographs taken from CTL and SAH animals showing Iba1-immunopositive cells around the branches of the middle meningeal artery in the dura mater. Magnified images of the selected area (dotted square) showing the greyscale pictures used for further analysis (lower panel). **b** Scatter plots showing four shape categories of Iba1-immunopositive macrophages delineated according to their shape attributes (circularity and roundness), in control (CTL) and after haemorrhage (SAH), indicating individual cells by different marks (circles and triangles) each assigned to their corresponding category. Bleeding reduced the proportion of elongated cells (from 30% to 20%), but increased the ratio of the amoeboid subgroup of mBAM (from 13% to 18%) in SAH animals relative to control. **c** Iba1-immunoreactive individual cells with different shape attributes (C: circularity, R: roundness, AR: major axis/minor axis) from the control dura preparation are presented as representative examples of the four corresponding macrophage categories. *n* = 952 and 1041, the total number of cells in CTL and SAH group that were analyzed

Three shape descriptors were determined: circularity, roundness, and elongation (the ratio of the major to the minor axis, AR), and 4 categories were identified: (1) circular with high circularity, (2) elongated with low circularity but high AR value, and two intermediate stages, (3) the amoeboid with higher roundness, and (4) intermediate, similar to the method of Travnickova et al. (2021). Depth-coding analysis revealed an increased number of amoeboid Iba1-immunoreactive cells in the upper tissue layers, whereas elongated cells were predominantly located in the deeper layers (Fig. 2d). Three days after SAH, a shift in the shape distribution of mBAMs was observed, with an increase in amoeboid cells and a decrease in elongated cells (Fig. 3). This unequal distribution of morphologically distinct cells suggests the migration of blood-derived Iba1-immunoreactive immune cells into the upper dural tissue within three days after bleeding.

**Fig. 4:**
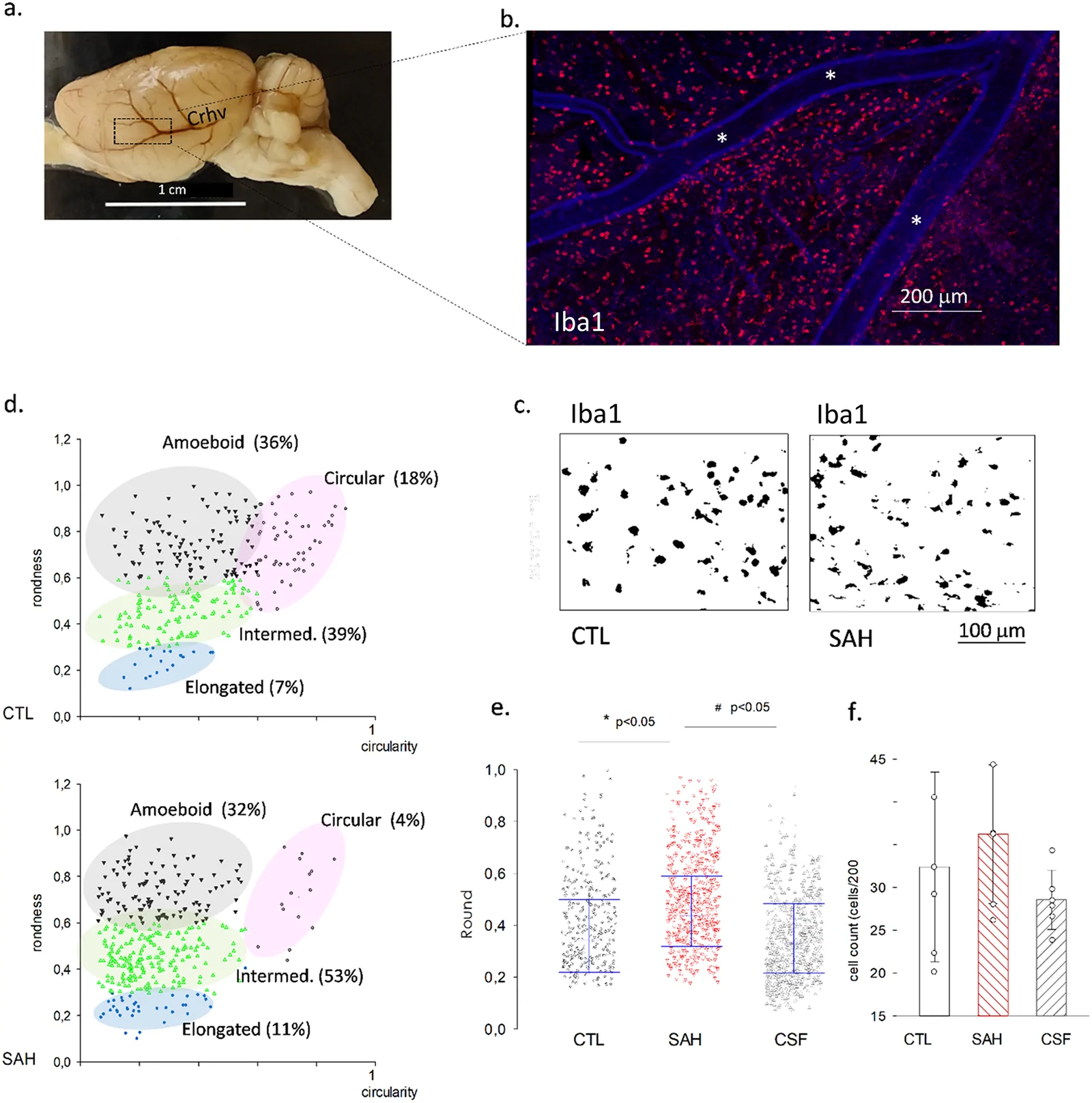
SAH induces a variation in the shape distribution of BAMs in the leptomeninges. **a** The photograph shows the area (black square) that was selected to make the leptomeningeal preparation. The rat brain was dissected with intact leptomeninges after transcardial perfusion with saline. Crhv: Caudal rhinal vein. **b** Representative photomicrographs taken from a control animal showing Iba1-immunopositive cells (red) in rat leptomeningeal preparation. Asterisks (*) indicate the lumen of the leptomeningeal blood vessels (blue). **c** Greyscale images of Iba1-immunostaining from control animals (CTL) and SAH animals (SAH) were used for morphometric analysis. The greyscale represents the intensity of the staining; Iba1-immunopositive cells are shown in black. **d** Scatter plots showing four shape categories of Iba1-immunopositive macrophages in leptomeninges delineated according to shape attributes such as circularity and roundness, in control (CTL) and after haemorrhage (SAH). Individual cells are indicated by different marks (circles and triangles) that sign their respective groups. Compared to the dura mater, leptomeninges contain fewer elongated but more amoeboid macrophages. The circularity of BAMs was reduced following bleeding as the proportion of circular cells decreased (18% vs. 4%, in CTL vs. SAH), but the proportion of the intermediate subgroup (Intermed.) increased in the SAH group relative to the control (53% relative to 39%). *n* = 361 and 530, the total number of cells in CTL and SAH group that were analyzed. **e** Morphometric analysis of meningeal macrophages indicates a decrease in the roundness of Iba1-immunopositive cells after SAH. Data were tested by Kruskal-Wallis One Way ANOVA on Ranks and pairwise comparisons were performed with Dunn’s post hoc test. The graph shows the roundness of individual cells in the CTL (black circle), SAH (red triangle), and CSF (grey triangle) groups, and the values are also expressed as median with range (IQR 25–75%) (blue lines) *: significant difference CTL vs. SAH; #: significant difference CTL vs. CSF. *n* = 304, 362 and 550, the total number of cells in CTL, SAH and CSF group that were analyzed. **f** Cell counting data have not revealed significant change in the number of Iba1-immunopositive cells in the leptomeninges after SAH. Data are expressed as mean ± S.D. and were analysed by One Way ANOVA followed by Fisher’s post hoc test. *n* = 6 in each group

In leptomeningeal preparations, the number of Iba1-immunopositive cells was not changed significantly after SAH (32 ± 4.53 vs. 36 ± 3.32 cells/200 × 200 μm, CTL vs. SAH), but morphometric data has revealed a change in their morphology (round: 0.61 vs. 0.5; *p* < 0.001 and circle: 0.55 vs. 0.19; *p* < 0.001; CTL vs. SAH) (Fig. 4). We have noticed a difference in the shape distribution of BAMs compared to the outer meningeal layer, the dura mater: higher number of amoeboid cells (36%) and a lower ratio of elongated cells in the control (7%). In contrast to the dural BAM population, a reduction in circularity of the total BAM population- a decrease in the proportion of circular cells- was detected after SAH (Fig. 4d). These data support that the subdural leptomeningeal BAM population differs in the reactive state compared to the macrophage population in the outermost meningeal layer, the dura mater encephali. The injection of CSF affects the morphology of Iba1-immunopositive cells in leptomeningeal preparations (round: 0.54 vs. 0.61, in the CSF vs. CTL group), but to a lesser extent than bleeding (11% decrease vs. 18% decrease after SAH) (Fig. 4e).

### Microglia activation following SAH in rat brain

**Fig. 5:**
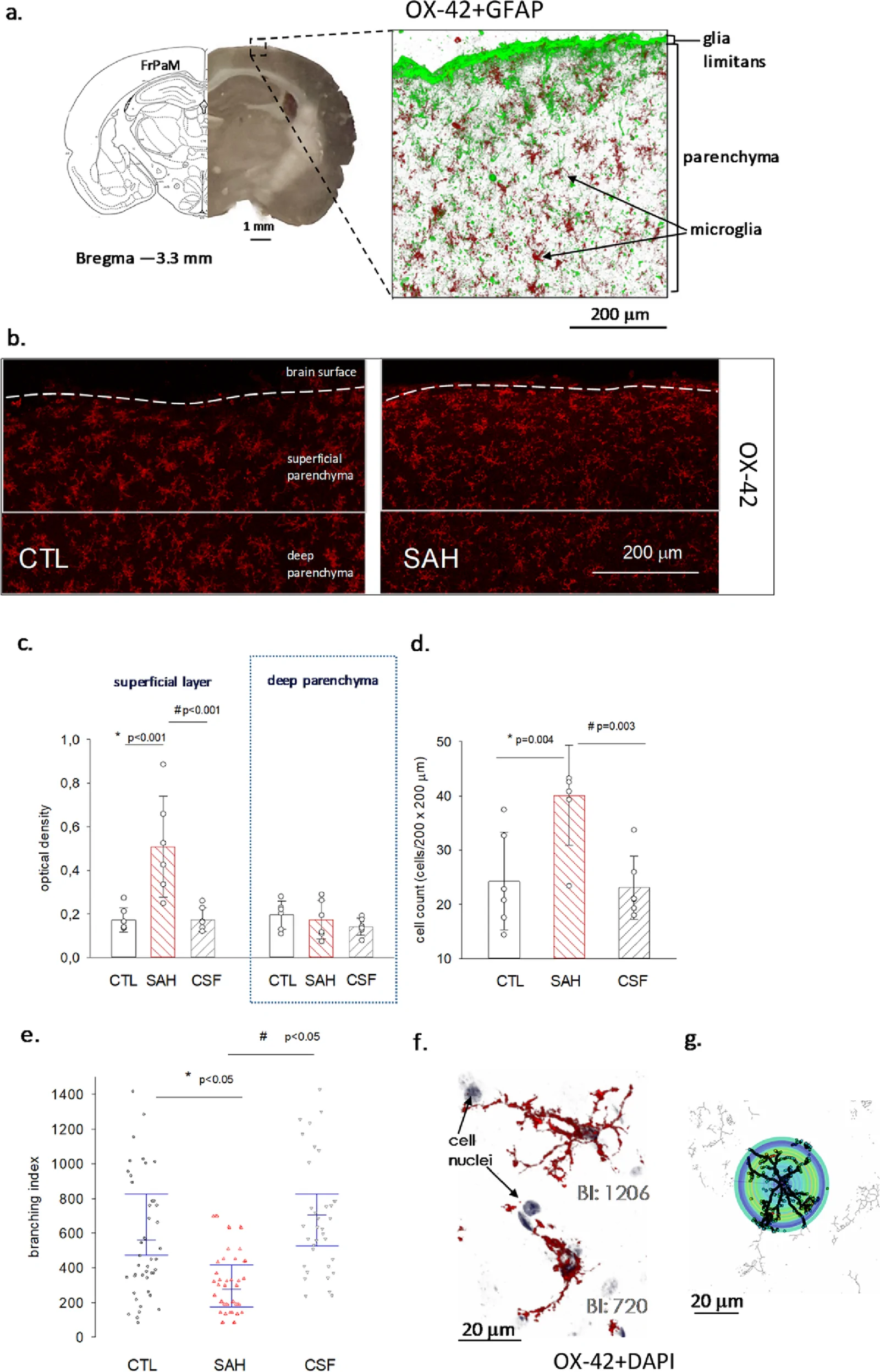
Microglia activation following SAH in rat brain. **a** The photograph shows a coronal section of the rat brain indicating a frontoparietal cortical area, which was selected for further analysis (black square). Right: Representative photomicrograph showing immunostaining with GFAP (green) and OX42 (red) in rat brain parenchyma. The GFAP labelling (green) on the brain surface represents the tight glia limitans layer formed by GFAP-immunopositive astrocytes, while the OX42-positive cells (red) are microglia. FrPaM: frontoparietal cortex. **b** Photomicrographs were taken from control (CTL) and SAH animal (SAH) showing OX42-immunostaining (red) in the upper motor cortex of rat selected for further analysis. The scale bar on the right refers to both images. **c** Increased OX42-staining intensity (OD) can be observed in the upper cortical region-, but not in the deeper parenchyma- in SAH animals relative to control groups (CTL, CSF). **d** The graphic shows an increase in the number of OX42-immunopositive cells for OX42-staining in the upper cortex after haemorrhage. **c** and **d** In the SAH group (SAH) the number of microglia and also the staining intensity (optical density) for the microglia marker OX42 increased relative to the control (CTL) or the cerebrospinal fluid injected group (CSF). Data are expressed as mean ± S.D. and were analysed by one-way ANOVA. Pairwise comparisons were made with Fisher post hoc test in each analysis. *: significant difference versus CTL, #: significant versus CSF. *n* = 6 in all groups. **e** SAH induces a significant decrease in microglia branching. Diagram shows the result of morphological analysis of OX42-labelled cells in the control (CTL), blood injected (SAH) and CSF injected (CSF) groups. The graph shows the branching index of individual cells in the CTL (black circle), SAH (red triangle), and CSF (grey triangle) group, and the values are also expressed as median with range (IQR 25–75%) (blue lines). Data were tested by Kruskal-Wallis One Way ANOVA on Ranks and pairwise comparisons were performed with Dunn’s post hoc test. *: significant difference vs. CTL; #: significant vs. CSF. CSF: cerebrospinal fluid. **f** Representative magnified (63x) image of OX42-labelled microglia with DAPI-labelled nuclei of surrounding cells (blue) and their respective shape attribute branching index (BI). **g** Representative image showing the Sholl analysis method performed to measure the branching tendency of individual microglial cells

Morphometric analysis of OX42-immunostaining enabled us to detect microgliosis in the rat brain 3 days after SAH. We evaluated the presence of OX42-immunopositive microglial cells by determining the optical density (OD) of OX42-staining (Fig. 5). An increased intensity of staining (OD: 0.17 ± 0.02 vs. 0.51 ± 0.09, CTL vs. SAH, *p* < 0.001) was observed near the surface of the parenchyma, in the upper cortical region of the parenchyma of the rat brain (OD: 0.19 ± 0.02 vs. 0.17 ± 0.03 in the CTL vs. SAH group), but not in deeper parenchymal regions suggesting polarisation of OX42-immunopositive microglial cells toward the surface (Fig. 5c). The increase in staining intensity is a consequence of the increase in the number of microglial cells in SAH group: 40 ± 3.76 cells/200 × 200 μm vs. 24 ± 3.65 cells/200 × 200 μm in CTL and 23 ± 2.37 cells/200 × 200 μm in CSF (*p* = 0.004 and *p* = 0.003, respectively) (Fig. 5d).

The reduction in process extension, as a response to inflammatory reactions, reflects the activation of microglia. 3 days after induction of bleeding, we detected a decrease in the branching tendency of OX42-immunolabeled cells relative to control and CSF (branching index, BI: 280 vs. 559 and 712, SAH vs. CTL and CSF) (Fig. 5e). Regarding the reactivity of microglia in the CSF group, neither the OX42 staining (OD: 0.14 ± 0.01) nor microglia morphology (BI: 712) showed significant differences compared to CTL, however, they were significantly different from SAH group (Fig. 5e). In contrast, no differences were observed in deeper parenchymal regions (OD: 0.17 ± 0.03 vs. 0.19 ± 0.02, SAH vs. CTL; not shown), indicating a preferential accumulation of OX42-positive microglia toward the parenchymal surface.

### SAH induces disintegration of the CNS border glia limitans layer

**Fig. 6:**
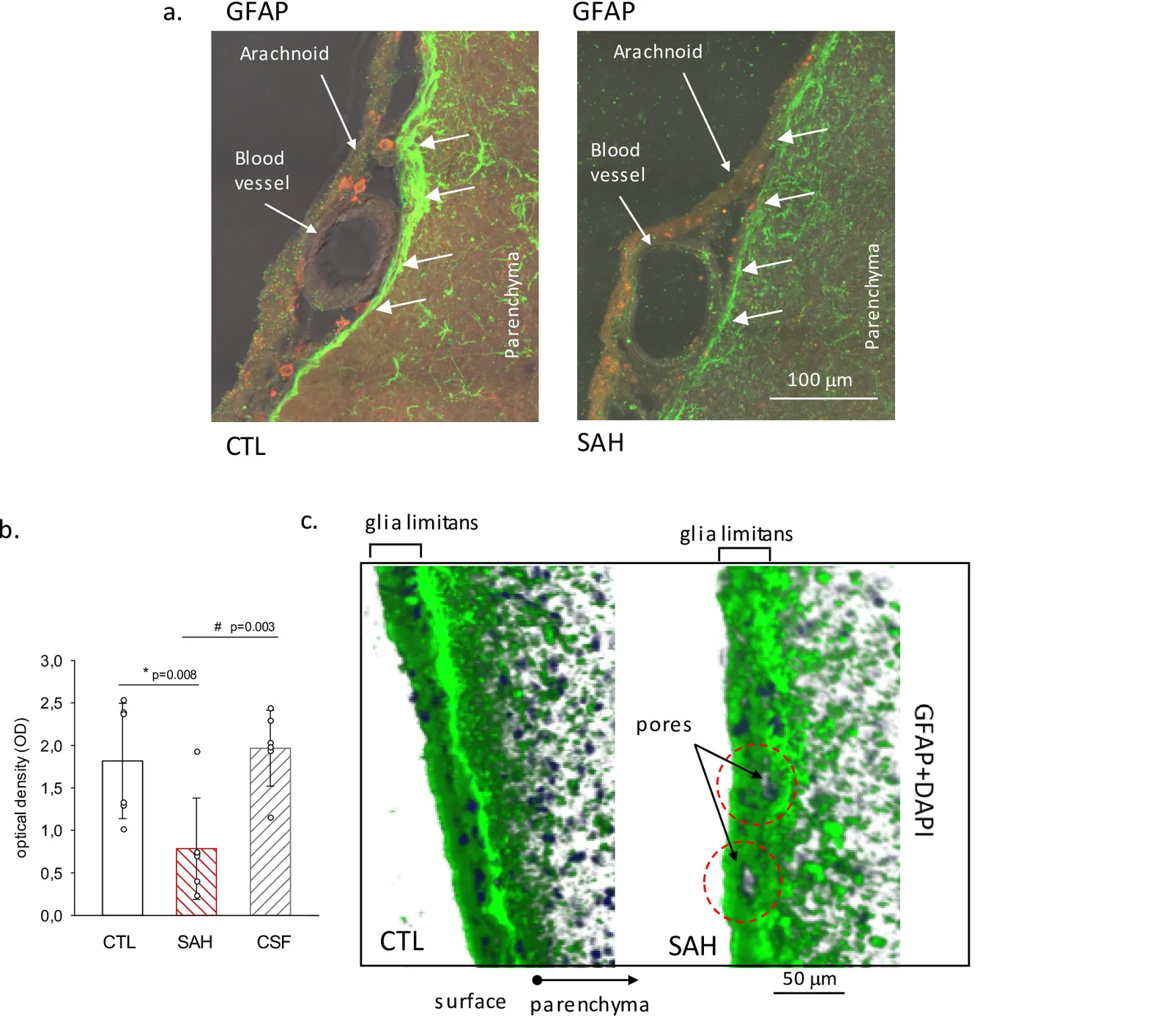
SAH induces disintegration of the glia limitans. **a** Photomicrographs taken from the coronal section of the frontoparietal cortex showing GFAP-immunopositive staining (green) on the surface of the rat brain in control (CTL) and after hemorraghe (SAH) with the attached leptomeningeal layer, the pia mater. Thinning of the GFAP-immunopositive layer was observed in SAH animals, indicating decreased GFAP-expression after haemorrhage. **b** The graph shows a decrease in the intensity (optical density) of GFAP-immunostaining on the parenchymal surface in the blood-injected group (SAH) relative to the control (CTL) or cerebrospinal fluid injected group (CSF). Data are expressed as mean ± S.D. and were analysed by one-way ANOVA. Pairwise comparisons were made with the Fisher post hoc test. *: significant versus CTL, #: significant versus CSF, *n* = 6 in all groups. **c** Three-dimensional images of GFAP-immunopositive glia limitans from a control (CTL) and SAH animal (SAH) with DAPI labelled cell nuclei (blue). Morphological observation detects pores (red dotted circles) in the SAH animal, indicating disintegration of the astrocyte-formed glial layer after bleeding

Antibody against the astrocyte marker GFAP was used to examine the glial barrier layer at the CNS border. GFAP-immunostaining revealed that SAH has a major effect on the integrity of glia limitans located at the parenchymal surface. A significant reduction in the density of GFAP-immunostaining suggests decreased expression of GFAP at the parenchymal-pial border in SAH animals (OD: 0.78 ± 0.24 vs. 1.82 ± 0.28, in SAH vs. CTL, *p* = 0.008) (Fig. 6a and b). We also examined GFAP staining in the deeper cortical region, where no reduction was detected after SAH (Fig. 6B Supplementary material). GFAP staining intensity is intrinsically higher at the glia limitans because astrocytic end-feet form a dense, continuous border in this region. Therefore, hemorrhage-induced reductions in GFAP immunoreactivity are more readily detected at the glia limitans and in the superficial cortex than in deeper cortical layer.

We also observed thinning of the glia limitans in the region of interest, since the average thickness of the GFAP-positive layer was 6.73 ± 0.94 μm in SAH animals, compared to 13.10 ± 1.06 μm in control animals (see Fig. 8a). Furthermore, the formation of pores in the GFAP-immunopositive layer was detected, demonstrating the astrocytic end foot disruption and the interruption of the continuity of glia limitans in SAH animals (Fig. 6c). We assessed GFAP intensity at the glia limitans at 8 h, 72 h, and 2 weeks after hemorrhage. Representative photomicrographs demonstrate increased GFAP staining during the acute phase of SAH, followed by a time-dependent decline, with no recovery at 2 weeks and evidence of glia limitans disorganization at the late stage (Fig. 6C Supplementary material). We therefore focused on the 72-hour time point for quantitative analysis in SAH animals, as it represents a transitional phase between early and delayed brain injury following subarachnoid haemorrhage.

After injection of CSF, the staining intensity remained similar to that of the control, but was significantly different compared to blood injection (OD: 1.97 ± 0.18 vs. 0.78 ± 0.24, CSF vs. SAH, *p* = 0.003) (Fig. 6b). These results provide morphological evidence for the destruction of barrier integrity and the subsequent increase in permeability of the CNS-border barriers in our SAH model.

### Impact of mast cells on SAH-induced glial reactions

#### SAH induces degranulation of meningeal mast cells

**Fig. 7:**
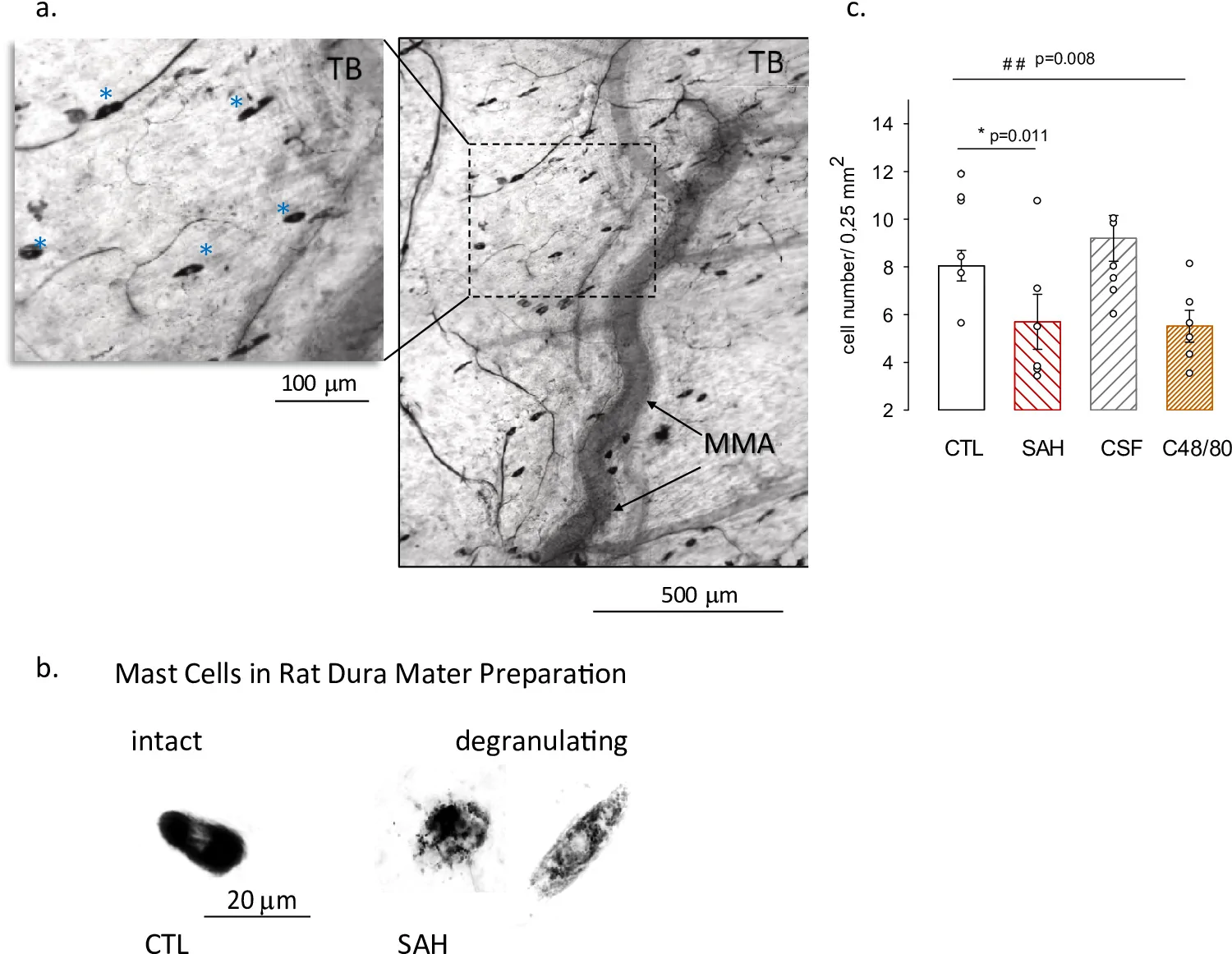
SAH induces mast cell degranulation in the dura mater of rats. **a** Photographs showing toluidine blue (TB) staining in dura mater whole-mount sample of control rat. For the counting and analysis of mast cells (blue asterisks), a 500 × 500 μm area was selected around the middle meningeal artery (MMA) (dotted square). **b** Representative samples of intact and degranulating mast cells from the control (CTL) and blood-injected animal (SAH). **c** SAH decreases the number of meningeal mast cells. The graph shows that the administration of the mast cell degranulator, C48/80, induced a similar reduction in the number of mast cells as haemorrhage. Data are expressed as mean ± S.D. and were analysed by one-way ANOVA. Pairwise comparisons with the Fisher post hoc test were made in each analysis. *: CTL was significant versus SAH, ##: CTL was significant versus C48/80, *n* = 6 in all groups. TB: toluidine blue, C48/80: compound 48/80

Mast cell (MC) degranulation was induced by systemic administration of compound 48/80 (C48/80), resulting in functional inhibition of MCs. This treatment was performed prior to the induction of subarachnoid hemorrhage (SAH) to block MC-mediated effects.Toluidine blue staining was performed to visualize mast cells in the meninges (mMCs) and to verify degranulation of mMCs after SAH (Fig. 7a, b). The number of stained cells in dura mater preparations isolated from CTL and CSF animals was not significantly different (9.2 ± 0.96 vs. 8.1 ± 0.64 cells/0.25 mm^2^, ±SEM), however, it was significantly reduced by approximately 40% in SAH animals compared to the control group (5.7 ± 1.15 vs. 9.2 ± 0.96 cells/0.25 mm^2^; ±SEM, *p* = 0.011) (Fig. 7c).

Despite their high numbers in the dura mater, MCs are not a particularly abundant type of immune cell in leptomeninges. Taking into account the relatively sparse presence of leptomeningeal MCs in CTL animals (0.3–0.6 cells/1 mm^2^), the number of MCs was not evaluated in the leptomeningeal preparations after SAH.

#### Depletion of mast cells attenuates adverse effects of SAH

Hereinafter, we studied how depletion of mMCs impacts neuroinflammatory reactions following SAH, such as the provoked activated state of microglia and impairment of the astrocyte boundary.

**Fig. 8:**
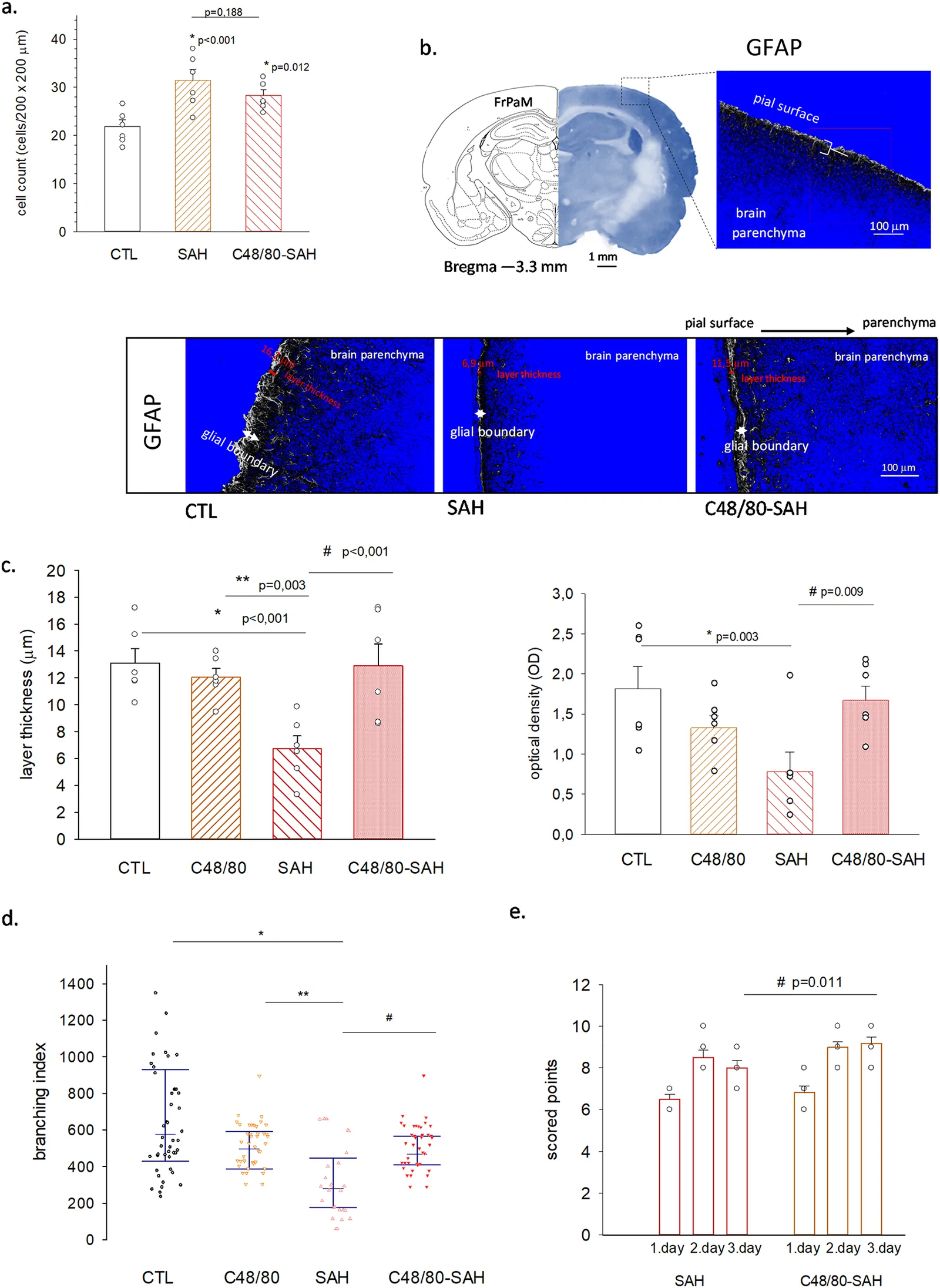
Depletion of mast cells attenuates the adverse effects of SAH. **a** Cell counting data indicate an increase in the number of Iba1-immunopositive cells in the rat dura mater after SAH when pretreated with C48/80. Data are expressed as mean ± S.D. and were analysed by One Way ANOVA followed by Fisher’s post hoc test. *: significant vs. CTL; *n*=6 in each group. **b** Photograph taken from coronal section of the rat brain showing the GFAP-immunopositive layer over the brain surface, and the underlying brain parenchyma. Red square: indicates the area selected for further analysis. The decrease in the number of mast cells (MC) ameliorates the damage to the astrocyte boundary. Representative photomicrographs showing the GFAP-immunopositive layer across the brain surface in the control, blood-injected (SAH) and compound48/80-pretreated SAH animal (C48/80-SAH). (The average thickness of the layer in µm is shown in red). **c** The graph (left) shows that haemorrhage-induced thinning of the GFAP-immunopositive boundary was prevented when mast cell depletion was induced before haemorrhage in the C48/80-SAH group. Administration of the mast cell degranulator compound48/80 (C48/80) did not alter the thickness of the GFAP layer compared to control. The graph (right) shows the decreased intensity of GFAP-immunostaining (optical density) in SAH group after haemorrhage. The results indicate that when the pretreatment with C48/80 preceded the induction of SAH (C48/80-SAH group), the staining intensity was similar to the control values (CTL), and was significantly improved compared to that measured in SAH. Data are expressed as mean ± S.D. and were analysed by one-way ANOVA. Pairwise comparisons with the Fisher post hoc test were made in each analysis. *: CTL was significant versus SAH, **: C48/80 was significant versus SAH and #: SAH was significant versus C48/80-SAH, *n*=6 in all groups. **d** The decrease in the number of mast cells moderates the SAH-induced microglia activation. The graph represents the results of the morphological analysis of OX42-labelled microglia showing the branching index of individual cells in the CTL (black circle), after administration of compound 48/80 (orange triangle), and in both haemorrhage groups (red triangle). Administration of compound 48/80 indicates a significant decrease in the microglial branching index after haemorrhage. The values are expressed as median with range (IQR 25-75%) (blue lines). Data were tested by Kruskal-Wallis One Way ANOVA on Ranks and pairwise comparisons were performed with Dunn’s post hoc test. *: significant difference vs. CTL; **: significant difference vs. C48/80 ; #: significant vs. C48/80-SAH. **e** Mast cell depletion improves the neurological outcome of SAH. The graph shows that haemorrhage caused significantly less neurological damage after compound48/80 pretreatment (C48/80-SAH group). Rats in the C48/80-SAH group improved their neurological scores on days 2 and 3 and their scores increased significantly compared to the animals in the SAH group. Data are expressed as mean ± S.D. and were analysed by One Way ANOVA followed by Fisher’s post hoc test. *: significant vs. CTL; *n*=6 in each group. A probability level of *p*<0.05 was considered statistically significant. #: significant difference SAH vs. C48/80-SAH. C48/80: compound48/80

First, quantitative morphometry was performed to verify whether a single injection of compound 48/80 (C48/80) achieved mast cell depletion in the meninges. Four days after C48/80 injection, the number of MCs decreased by 40% in the dura mater preparations (from 9.2 ± 0.96 to 5.5 ± 0.66 cells/0.25 mm^2^) (Fig. 7c). Subsequently, SAH was induced in a subset of pretreated animals (C48/80-SAH group) and additional morphometrical analysis was performed to clarify the role of mMC depletion in consecutive macrophage and glial reactions. Regarding mBAM activation, the number of Iba-immunopositive cells was compared between the SAH and the C48/80-SAH group in the preparation of the dura mater. According to cell count data, injection of blood increased the number of mBAMS in C48/80-SAH animals (by 27% from 22 ± 1.38 to 28 ± 1.16 cells/200 × 200 μm, in CTL vs. C48/80-SAH, *p* < 0.012), but to a slightly lower extent than without pretreatment with C48/80 (28 ± 1.16 vs. 31 ± 5.48 cells/200 × 200 μm, SAH vs. C48/80-SAH, *p* = 0.188) (Fig. 8a). Subsequently, the SAH-induced subsequent glial morphological alterations were examined. When C48/80 pretreatment preceded induction of SAH, the optical density and also the thickness of the GFAP immunopositive layer were both similar to control values, but significantly differed from those measured after SAH without pretreatment (OD: 1.66 ± 0,27 vs. 0.78 ± 0.24, C48/80-SAH vs. SAH, and layer thickness: 12.90 ± 1.63 vs. 6.73 ± 0.94 μm, in C48/80-SAH vs. SAH) (Fig. 8b and c). Furthermore, in the C48/80-SAH group, the branching index of microglial cells did not show significant differences relative to CTL, however, it was significantly differed from the value measured after haemorrhage in the SAH group (BI: 467 vs. 280, in C48/80-SAH and SAH) (Fig. 8d). These results suggest that the decrease in the number of MCs moderates SAH-induced microglia activation and also ameliorates SAH-induced damage to the astrocyte boundary.

Furthermore, in accordance with immunohistochemical results, we detected that MC depletion improves neurological outcome of SAH. Induction of SAH caused a significant decrease in neurological functions, but SAH caused significantly less neurological damage after C48/80 treatment. Rats in the C48/80-SAH group improved their neurological scores on days 2 and 3 (9.0 ± 0.25 and 9.17 ± 0.30 points, respectively) and their scores became significantly higher compared to the animals in the SAH group (Fig. 8e).

## Discussion

In the present study, we demonstrated that SAH induces activation of immune cells associated with the CNS border, contemporaneously with the early neuroinflammatory reactions that take place in the parenchyma.

First, we characterised the border-associated macrophages as the major immune cells in the meningeal tissues. A previous study performing in vivo analyses reported the existence of four morphological shapes of migrating macrophages (Travnickova et al. 2021). Based on high resolution microscopic imaging and macrophage shape descriptor analysis we have identified four morphological subgroups of meningeal macrophages: amoeboid, circular, intermediate, and elongated shape. Following SAH, we discovered a change in the shape distribution of mBAMs: an increased ratio of circular cells and a decreased proportion of the elongated population, reflecting a migration process after haemorrhage. Previous studies have demonstrated that mBAMs can be replenished by recruiting blood-derived or bone marrow-derived monocytes in inflammatory conditions (Cugurra et al. 2021). Cell counting data and depth code analysis support the invasion of myeloid cells in the dura mater.

The subdural meninges harbour a particular immune cell population, with peculiar macrophages and microglia (Frosch and Prinz 2025). Consistent with that, we found that macrophages exhibit a distinct shape distribution in the soft and hard meninges, and SAH affects the distribution differently. Morphometrical analysis suggests dynamics and morphological plasticity of residual macrophages in the leptomeninges, rather than recruitment.

Neuroinflammation, a well-defined consequence of SAH, is a prominent cause of early brain damage (Zheng and Wong 2017; Wang et al. 2020). Concomitant with the activation of meningeal macrophages, we reported the prevalence of neuroinflammatory reactions, including the microglial reaction and the loosening of the glial barrier. Morphological analysis revealed enhanced activation of microglia in the fronto-parietal cortex of rats after induction of SAH. A limitation of the present study is that microglial activity was evaluated using the branching index as a morphological surrogate of activation, reflecting changes in process complexity. While this provides useful information on structural alterations, it does not directly assess molecular or functional activation states. Future studies incorporating specific activation markers such as CLEC7A may therefore further strengthen the characterization of microglial responses.

We demonstrated further morphological evidence for the decay of the astrocyte border glia limitans on the surface of parenchyma. Morphological analysis of the GFAP-immunopositive boundary layer revealed thinning of the barrier and interruption of continuity through the formation of pores within it. Studies of the BBB suggest that under inflammatory conditions, when the BBB becomes more permeable, diffusion of solutes and also passage of immune cells such as MCs may occur (Marchetti and Engelhardt 2020; Silverman et al. 2000). Consistent with these studies, our results confirmed the loosening of CNS barriers after SAH and support the presumed trafficking of immune cells through the CNS boundaries.

A limitation of the present study is that blood–brain barrier integrity and functional permeability changes were not directly assessed. While our focus was on glial and meningeal responses at the level of the parenchymal surface and glia limitans, future tracer-based studies will be important to investigate meningeal–arachnoid–CSF–parenchymal exchange following subarachnoid hemorrhage, including the movement of larger molecules between these compartments.

Previous studies documented mast cell activation early after a stroke event (Strbian et al. 2006) and highlighted the significance of MCs as critical regulators of BBB changes in the pathogenesis of several CNS diseases (Ribatti 2015; Lakatos and Rosta 2025; Kothari et al. 2025). We also found that dural MC degranulation is associated with the SAH event. In parallel, the number of countable cells decreased, probably because of a reduced number of visible granules as a result of degranulation. Furthermore, we confirm the crucial role of MC activation in the pathomechanism of SAH early after haemorrhage. We showed that systemic administration of C48/80 resulted in a marked decrease of dural MCs, and the subsequent depletion of MCs affects neuroinflammatory reactions after SAH. Branch retraction, indicative of microglial activation, was impeded when C48/80 was applied before SAH. Additionally, the integrity of the astrocyte boundary was retained in SAH following C48/80 pretreatment. Parallel with glial reactions, we also showed that pretreatment with C48/80, however, did not prevent the increase in the number of meningeal BAMs following SAH, suggesting that BAMs do not play a crucial role in subsequent glial reactions. While recent transcriptomic studies have explored the role of the dura mater in SAH response (Zhu et al. 2025), they do not report alterations in mast cells or the glia limitans, which are specifically addressed here.

In addition, neurobehavioural tests were performed to determine the clinical manifestation of haemorrhage in our single injection model. Neuroinflammation has been reported to play a key role in the development of cognitive impairment in various neurological diseases Gan et al. 2025). Therefore, we hypothesised that the extent of neuroinflammation associated with SAH significantly influences the disease outcome. Consistent with the morphological results, significant neurological impairment was demonstrated after SAH, however, depletion of MCs improved the neurological outcome of the haemorrhage.

Our results are consistent with previous studies supporting the importance of the meningeal immune niche in certain neurological disorders, such as stroke or SAH (Tan et al. 2024). The strategic location of mBAMs and mMCs determines the immune surveillance of the submeningeal and parenchymal milieu. Consequently, an in-depth understanding of activation, migration, and site infiltration is important for the therapeutic approach. Recently, it has been shown that macrophage depletion results in impaired outcomes reflecting the importance of BAMs in the pathogenesis of SAH (Wan et al. 2021). Based on our data, we concluded that simultaneous activation of mBAMs and mMCs and the parenchymal glial cells presents a possibility for bidirectional communication, especially due to the loosening of the barriers. Expanded morphological and functional investigation of the CNS-meningeal border as a communication interface would promote the development of new immunotherapeutic strategies after haemorrhage.

## Key points

In our manuscript entitled “Simultaneous Activation of Border-associated Immune Cells and Glial Cells at the CNS-meningeal Interface after Subarachnoid Haemorrhage in Rats”,

- we performed the morphological characterisation of the border-associated macrophage subpopulation in the dura mater and leptomeninges of rats,
- we demonstrated the activation of immune cells associated with the CNS border, contemporaneously with the early neuroinflammatory reactions that occur in the brain parenchyma after experimental subarachnoid haemorrhage (SAH),
- we provide morphological evidence that SAH substantially affects the integrity of the CNS boundaries, especially the astrocyte barrier ‘glia limitans’,
- and we confirmed the crucial role of mast cells in the subsequent decomposition of the glial boundary.

## Supplementary Information

Below is the link to the electronic supplementary material.

Supplementary Material 1

Supplementary Material 2

Supplementary Material 3

## Data Availability

Data cannot be shared openly but are available on request from authors.

## Abbreviations

BAM: Border-associated macrophage
CNS: Central nervous system
CSF: Cerebrospinal fluid
GFAP: Glial fibrillary acid protein
Iba-1: Ionised calcium binding adaptor-1 molecule
IR: Immunoreactive
MC: Mast cell
mMCs: Meningeal mast cells
mBAMs: Meningeal border-associated macrophages
MMA: Middle meningeal artery
SEM: Standard error of the mean
SIF: Synthetic interstitial fluid
SAH: Subarachnoid haemorrhage
TB: Toluidine blue
