# Supplementary figures and images for: Simultaneous activation of border-associated immune cells and glial cells at the CNS-meningeal interface after subarachnoid haemorrhage in rats
Source: Brain Struct Funct. 2026 Jul 22;231(7):105. doi: 10.1007/s00429-026-03156-y (PMC13391711; doi:10.1007/s00429-026-03156-y)

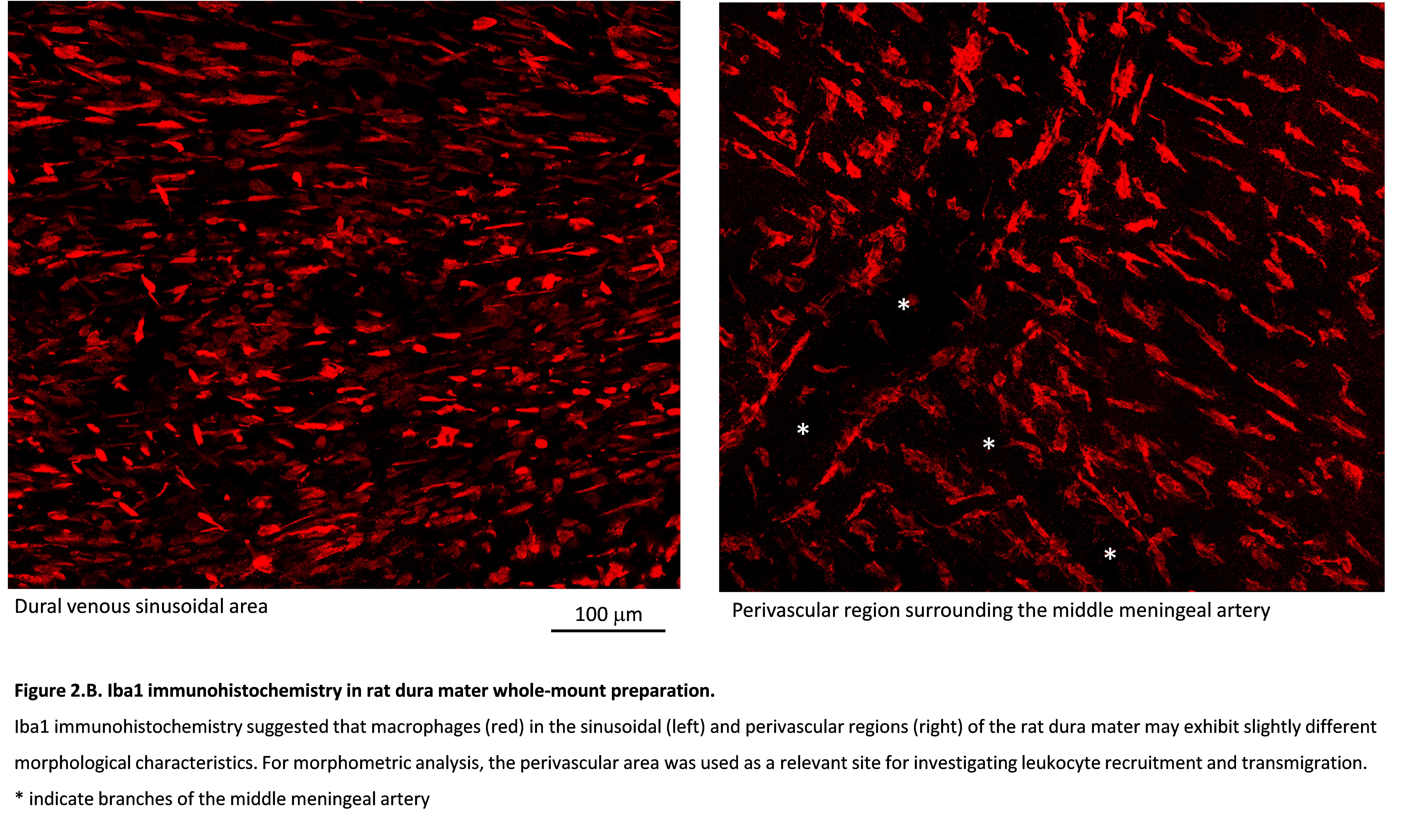

Supplement: Supplementary file 1 — Supplementary Material 1 [file 429_2026_3156_MOESM1_ESM.tif]

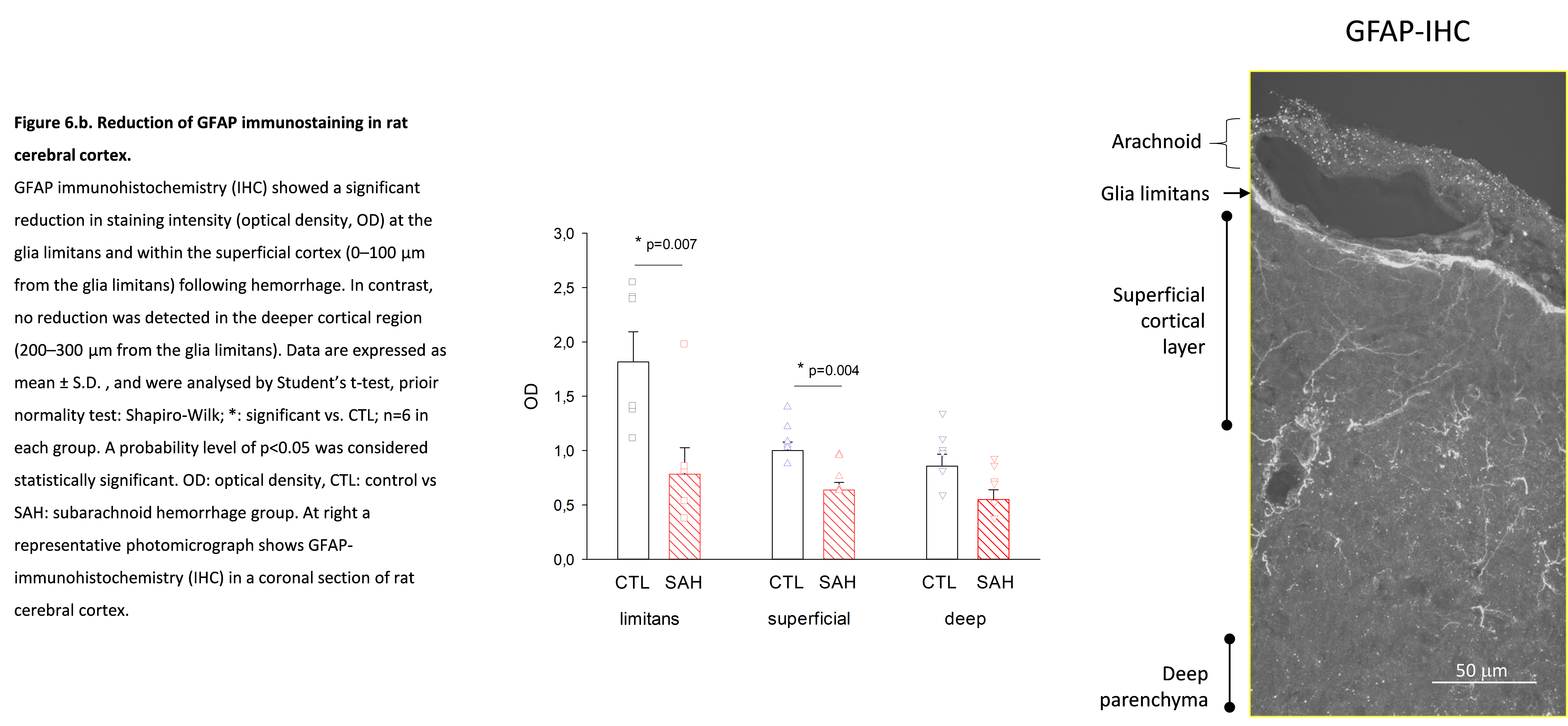

Supplement: Supplementary file 2 — Supplementary Material 2 [file 429_2026_3156_MOESM2_ESM.tif]

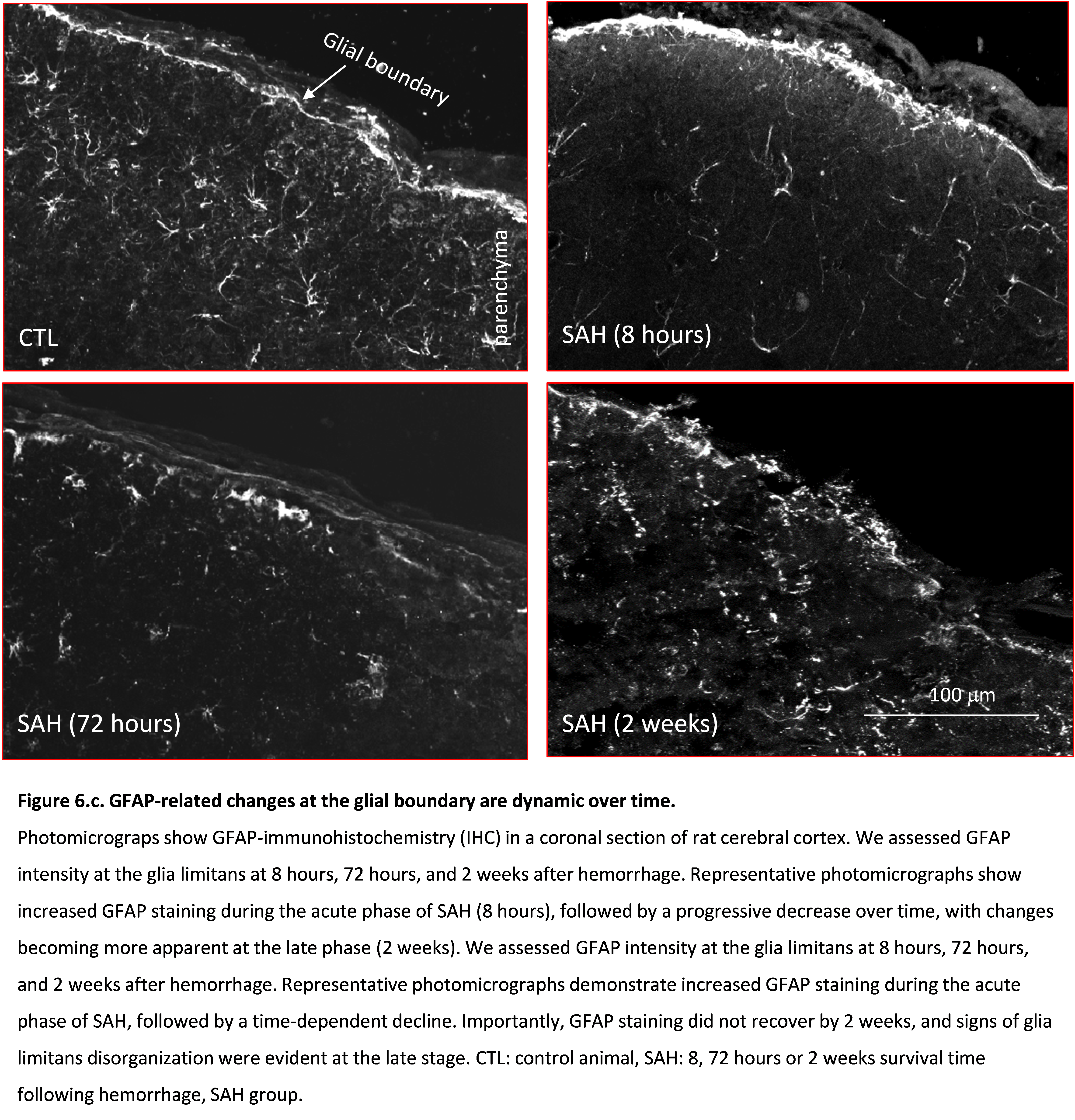

Supplement: Supplementary file 3 — Supplementary Material 3 [file 429_2026_3156_MOESM3_ESM.tif]
